# Formyl peptide receptor 2 activation by mitochondrial formyl peptides stimulates the neutrophil proinflammatory response via the ERK pathway and exacerbates ischemia–reperfusion injury

**DOI:** 10.1186/s11658-023-00416-1

**Published:** 2023-01-19

**Authors:** Yirui Cao, Juntao Chen, Feng Liu, Guisheng Qi, Yufeng Zhao, Shihao Xu, Jiyan Wang, Tongyu Zhu, Yi Zhang, Yichen Jia

**Affiliations:** 1 Department of Urology, Zhongshan Hospital, Fudan University, Shanghai, China; 2 Shanghai Key Laboratory of Organ Transplantation, Shanghai, China; 3 Zhongshan Hospital Institute of Clinical Science, Zhongshan Hospital, Fudan University, Shanghai, China; 4 Department of Integrative Medicine, Huashan Hospital Fudan University, Shanghai, People’s Republic of China

**Keywords:** Formyl peptide receptor 2, Neutrophil, Kidney IRI, Migration, Mitochondrial-derived formyl peptides

## Abstract

**Background:**

Ischemia–reperfusion injury (IRI) is an inevitable process in renal transplantation that significantly increases the risk of delayed graft function, acute rejection, and even graft loss. Formyl peptide receptor 2 (FPR2) is an important receptor in multiple septic and aseptic injuries, but its functions in kidney IRI are still unclear. This study was designed to reveal the pathological role of FPR2 in kidney IRI and its functional mechanisms.

**Methods:**

To explore the mechanism of FPR2 in kidney IRI, the model rats were sacrificed after IRI surgery. Immunofluorescence, enzyme-linked immunosorbent assays, and western blotting were used to detect differences in the expression of FPR2 and its ligands between the IRI and control groups. WRW_4_ (WRWWWW-NH2), a specific antagonist of FPR2, was administered to kidney IRI rats. Kidney function and pathological damage were detected to assess kidney injury and recovery. Flow cytometry was used to quantitatively compare neutrophil infiltration among the experimental groups. Mitochondrial formyl peptides (mtFPs) were synthesized and administered to primary rat neutrophils together with the specific FPR family antagonist WRW_4_ to verify our hypothesis in vitro. Western blotting and cell function assays were used to examine the functions and signaling pathways that FPR2 mediates in neutrophils.

**Results:**

FPR2 was activated mainly by mtFPs during the acute phase of IRI, mediating neutrophil migration and reactive oxygen species production in the rat kidney through the ERK1/2 pathway. FPR2 blockade in the early phase protected rat kidneys from IRI.

**Conclusions:**

mtFPs activated FPR2 during the acute phase of IRI and mediated rat kidney injury by activating the migration and reactive oxygen species generation of neutrophils through the ERK1/2 pathway.

**Supplementary Information:**

The online version contains supplementary material available at 10.1186/s11658-023-00416-1.

## Background

Ischemia–reperfusion injury (IRI) is the main cause of delayed graft function (DGF) in kidney transplant patients. According to a US statistic that included more than 50,000 patients, DGF occurred in at least 23% of kidney transplant patients [1]. DGF can not only extend hospitalization time but can also bring permanent harm to the transplanted kidney, including higher risks of graft rejection, tissue fibrosis, and even graft loss [2]. Although numerous meaningful studies have been conducted, the mechanism underlying kidney IRI is still elusive [3]. Among the multiple mechanisms of injury, metabolic abnormalities, oxidative stress, and neutrophil- and macrophage-related damage are generally recognized as key injury mechanisms [4].

In recent years, the importance of formyl peptide receptors (FPRs) in sterile inflammation has drawn much attention. In the human body, the FPR family consists of three members: FPR1, FPR2, and FPR3, which are normally found on the surface of myeloid immune cells such as neutrophils and macrophages [5]. The polypeptides recognized by FPRs share a common molecular feature: a formylated N-terminus. This feature is unavoidable during the translation of bacterial proteins. Mitochondrial proteins also exhibit the N-terminal formylation feature, likely because mitochondria share a common origin with bacteria. Proteins and peptides with this structural feature are recognized by FPRs as pathogen-associated molecular patterns (PAMPs) or damage-associated molecular patterns (DAMPs), marking an invasion of bacteria (bacterial formyl peptides) or destruction of mitochondria (mitochondrial formyl peptides, mtFPs). Among these FPRs, FPR2 is the most intriguing receptor because of its multiple downstream functions and numerous upstream agonists [6]. In addition to formylated peptides, FPR2 recognizes proteins and lipids as internal agonists and exhibits pro- or anti-inflammatory functions depending on the ligand that activates it. FPR2 plays important roles in a variety of sterile inflammatory and autoimmune diseases [6]. In rheumatoid arthritis, knockdown of FPR2 on the surface of leukocytes increases the extent of tissue damage [7]. However, in chronic obstructive pulmonary disease, FPR2 is activated by its proinflammatory ligands, which activate leukocyte migration and exacerbate inflammation [8]. In recent years, the role of FPR2 in intrinsic immunity has increasingly been recognized as important [8, 9]; however, FPR2 has seldom been directly investigated in kidney IRI. The natural pathology of FPR2 in kidney IRI is still elusive.

Neutrophils are one of the major effector cells in the intrinsic immune system. The classical immune functions of neutrophils include migration ability, phagocytosis, reactive oxygen species (ROS)-dependent and non-ROS-dependent killing effects, and ability to release inflammatory factors [10]. These functions are the biological basis by which neutrophils kill pathogens, remove necrotic cells, and damage adjacent tissue [11]. In addition to their antimicrobial function, neutrophils also play an important role in a variety of sterile inflammatory conditions, including ischemia–reperfusion injury, acute respiratory distress syndrome, tumors, and other conditions [12–14]. Under these sterile conditions, neutrophil migration capacity, ROS-dependent and non-ROS-dependent killing effects, and inflammatory factor release capacity are strongly associated with neutrophil-induced damage to healthy tissue. FPR2 is expressed on the surface of neutrophils and can mediate different or even opposite important immune functions in certain diseases [15, 16]. However, there is a lack of mechanistic studies on the function of neutrophil FPR2 in IRI.

In many disease models, including hyperoxygen-induced lung injury, conjunctive goblet cells, and influenza infection models, FPR2 activates the ERK1/2 pathway to trigger downstream immune functions [17–19]. ERK1/2 is a classical signaling pathway that transduces extracellular signals into the cell. ERK1/2 is mainly activated by signals from various cytokines and membrane receptors and conducts activation signals into the cell, activating downstream transcription factors, such as c-myc, c-jun, and NF-κB, to alter the expression levels of specific genes [20]. ERK1/2 is also a common transduction pathway downstream of the FPR family [21]. However, this is not always the case. In Crohn’s disease, macrophage FPR2 can direct neutrophil function by activating the PI3K/Akt pathway [22]. It is not yet known whether ERK1/2 mediates the signaling generated by FPR2 activation in IRI. Here, we aimed to further explore the exact pathological role of FPR2 in kidney IRI, looking for clues related to the treatment of IRI. Furthermore, we aimed to investigate the role of FPR2 in the acute phase of IRI and explore how it is regulated by the ERK1/2 pathway.

## Methods

### Animals, ischemia–reperfusion injury model and treatment

Six-week-old Sprague–Dawley rats (weighing 150–200 g) were purchased from Shanghai JieSiJie Laboratory Animal Co. Ltd and bred in a specific-pathogen-free (SPF)-grade environment in the experimental center of Zhongshan Hospital.

Rats were randomly assigned to three groups: (1) sham operation group: abdominal cavity of the anesthetized rats were exposed for 45 min; (2) ischemia–reperfusion group: after anesthetization, the right kidney was removed, and pedicle of the left kidney was clamped for 45 min; (3) WRW_4_ treatment group: rats were intravenously injected with WRW_4_ (MedChem Express, Monmouth Junction, NJ, USA) solution at a dose of 10 mg/kg slowly 30 min before kidney IRI was performed. Animals were kept on heating pad to keep body temperature before awaken. Twenty-four hours after surgery, these rats were sacrificed to harvest tissue and blood samples.

### Primary cell isolation, culture and treatment

Femur and tibia bones of rats were harvested and flushed inside bone marrow cavity by sterile PBS for bone marrow cells. Red blood cell lysis buffer (Absin Bioscience Inc., SH, China) was used to lyse red blood cells. Remaining cells were isolated by a density gradient centrifugation base on Percoll (Absin). Density of Percoll layers was respectively configurated to 52% (Percoll:PBS, 1: 2), 69% (Percoll:PBS, 1: 1), and 78% (Percoll:PBS, 6: 1). Cell layer between 52% and 69% Percoll layer that consisted mainly of neutrophils was collected and washed with RPMI 1640 medium (GIBCO BRL, Grand Island, NY, USA) three times for further experiments. Neutrophils were cultured by pure RPMI1640. All cell experiments were performed within 24 h after animal sacrifice.

### Renal function assay

Peripheral blood was aspirated from the left ventricle of the anesthetized rats and kept in EDTA-rinsed 15 ml centrifuge tubes cooled by ice. Whole blood was centrifuged at 3000*g* at 4 °C for 10 min. Plasma was then separated from blood cells for further testing. Serum creatine and blood urea nitrogen were measured by QuantiChrom Creatinine Assay Kit and QuantiChrom Urea Assay Kit (BioAssay Systems, Hayward, CA, USA) according to the manufacturer’s product instructions.

### Enzyme-linked immunosorbent assay (ELISA)

Cytokines, Annexin A1 (ANXA_1_), Resovin D1 (RvD1), Lipoxin A4 (LXA_4_), serum amyloid A (SAA), and NADH dehydrogenase subunit 6 (ND_6_) in serum and kidney samples were measured using commercially available ELISA kits (MLBio, SH, China). Experimental procedures were performed as per the manufacturer’s instructions. Kidney tissue samples were homogenized in PBS. Protein concentration in the sample was quantified by bicinchoninic acid (BCA) method and then diluted to the same total protein concentration using PBS before ELISA measurement.

### RNA isolation and quantitative reverse transcription PCR (RT-qPCR)

Kidney tissue was homogenized in TRIzol reagent (Sigma-Aldrich (SH, China) Trading Co., Ltd) for total RNA extraction. RNA isolation procedures were performed as the manufacturer’s product instructions. Quality of RNA was assessed by A260/A280 ratio. Reverse transcription was performed using first-strand cDNA reverse transcription kit (Vazyme, NJ, China). RT-qPCR was performed in duplicate using Hieff qPCR SYBR Green Master Mix (No Rox) (Yeasen, SH, China). An ABI QuantStudio5 platform was used for the qPCR protocol. Threshold values were corrected by β-actin expression as an endogenous control. Primer sequences were designed by Primer3Plus and verified by NCBI BLAST (Table 1).

**Table 1:** Primers used for qPCR

Genes	Primers
IL-6	Forward: 5′-TGAACAGCGATGATGCACTG-3′ Reverse: 5′-AGAAACGGAACTCCAGAAGACC-3′
TNFα	Forward: 5′-ACGATGCTCAGAAACACACG-3′ Reverse: 5′-AAGCCCATTGGAATCCTTGC-3′
IFN-γ	Forward: 5′-ATCGAATCGCACCTGATCAC-3′ Reverse: 5′-TTGGCGATGCTCATGAATGC-3′

### Hydrogen peroxide (H_2_O_2_), malondialdehyde (MDA), and superoxide dismutase (SOD) assay

Cell supernatant was collected and measured immediately using H_2_O_2_ assay kit (Beyotime Biotechnology, SH, China). Kidney tissue oxidation was measured by MDA and SOD using commercially available kits (Elabscience Biotechnology Co.,Ltd, WH, China). Experiments of these assays were performed according to the manufacturers’ instructions.

### Western blotting

Kidney tissue was stored temporally by a mixture of RIPA, PMSF, and phosphatase inhibitor before homogenization. Tissue lysate was then centrifuged at 12,000*g* at 4 °C for 15 min. The supernatant was separated and quantified by BCA method. After sample preparation, proteins were separated by SDS–PAGE and transferred to polyvinylidene fluoride membrane. The membranes were blocked with 5% milk for 2 h and incubated overnight with 1:1000 diluted primary antibodies, including anti-β-actin (Yeasen), anti-ANXA_1_ (Abcam, Cambridge, MA, USA), anti-SAA (Thermo Fisher Scientific, Waltham, MA, USA), anti-MMP-9 (Abcam), anti-FPR2 (Novus Biologicals, CO, USA), anti-tERK1/2 (Cell Signaling Technology, Danvers, MA, USA), anti-pERK1/2 (Cell Signaling Technology), and anti-GAPDH (ABclonal Technology Co., Ltd, WH, China). The membranes were incubated with 1:3000 diluted goat-derived anti-rabbit IgG (Abclonal) for 1.5 h and detected by chemiluminescence reagents (Merck Millipore, Billerica, MA, USA).

### Single-cell suspension preparation and flow cytometry

Kidney tissue was dissected into small pieces and gently minced by gentleMACS Dissociator (Miltenyi Biotec GmbH, Bergisch Gladbach, Germany) in Hank’s buffer containing 10% type IV collagenase (Gibco). Red blood cells were lysed in both kidney and blood samples. Single cells were divided into groups and stained by fluorescence-conjugated antibodies including CD45-APCCy7 (BD Pharmingen, San Diego, CA, USA), CD11b-APC (BD), FPR2-AF647 (Bioss, Beijing, China), CD86-BB700 (Novus), CD163-AF405 (BD), and CD62L-BV421 (BD) for 30 min in staining buffer (BioLegend, San Diego, CA, USA). Neutrophil Elastase-PE (Bioss), MPO-FITC (BioLegend), and CD68-PE (Invitrogen) were stained after fixation/permeabilization using fixation and fixation/permeabilization buffer (BioLegend) according to the manufacturer’s protocols. Apoptosis assay was conducted using commercially available Annexin V/PI staining kit (Beyotime) according to the manufacturer’s instructions. Experimental data were acquired using FACS Aria III (BD Biosciences, San Jose, CA, USA) and analyzed by Flow Jo X (BD Biosciences).

### Transwell assay

Migratory ability of neutrophils was evaluated by 3-µm-pore-sized transwell inserts (Corning Incorporated, Corning, NY, USA). Bottom chambers were filled with pure RPMI1640 medium dissolved with mtFP, mtFP and WRW_4_, and mtFP and SCH772984 (MedChem Express). A total of 1 × 10^5^ cells were seeded in the top chamber with serum-free RPMI1640 medium, and cultured in 5% CO_2_ at 37 °C for 2 h. Culturing plates were then centrifuged at 1500*g*, 4 °C for 5 min to disassociate cells attached beneath the membrane- and sediment-suspended cells. The top chamber was then removed. The number of cells in every bottom chamber was counted for five fields under a light microscope for statistical analysis.

### MtFP preparation

Mitochondrial peptide was synthesized according to ND_6_ N-terminal sequence (N-formyl-Met-Met-Tyr-Ala-Leu-Phe) [23] and dissolved into PBS.

### Histopathology

Hematoxylin and eosin (H&E) staining was performed to evaluate the severity of kidney injury. Tissue sections magnified by tenfold were blind-labeled and evaluated by two independent pathologists. Kidney injury was assessed by the damage percentage of tubular cells: 0, none; 1, ~ 0 –11%; 2, ~ 11–25%; 3, ~ 26–45%; 4, ~ 46–75%; 5, 75%. Typical damages included proximal tubular cell brush border loss, swelling or vacuolization, cell necrosis, and formation of casts. Higher score indicated a more severe kidney injury.

### Immunohistochemistry analysis

Immunohistochemical staining was performed on paraffin sections. Antigen retrieval was performed by EDTA (pH 9.0) in steam bath maintained by microwave. Sections were washed with TBS three times after cooling, and then incubated in 3% H_2_O_2_ for 30 min. Antigen was blocked by 10% goat serum for 30 min. The sections were labeled with primary antibodies at 4 °C overnight. Goat-anti-rabbit antibody was added to the sections on the next day. DAB was used to reveal antibody binding states. Prepared slides were photographed by ECLIPSE E600 microscope with an attached Digital Sight Camera (Nikon, Tokyo,Japan).

### Immunofluorescence staining

Rabbit anti-FPR2 antibody (Novus), mouse anti-CD68 (Abcam) and mouse anti-MPO (Abcam) were used as primary antibodies and were separately detected by Alexa Fluor 488 donkey anti-rabbit secondary antibody (1:1000; Invitrogen) and Alexa Fluor 594 donkey anti-mouse antibody (1:200, Invitrogen). For nuclear staining, 4′,6-diamidino-2-phenylindole (DAPI) was used.

### Terminal deoxynucleotidyl transferase dUTP nick end labeling (TUNEL) assay

To examine the apoptosis of tubular cells in kidney, TUNEL assay was applied using an in situ cell death detection kit (Roche, Basel, Switzerland) and processed according to the manufacturer’s protocols.

### Statistical analysis

Data were analyzed using GraphPad Prism 8. Quantitative data were analyzed by two-tailed independent *t*-test and expressed as mean ± SD. *P* < 0.05 was considered as statically significant.

## Results

### Specific inhibition of elevated FPR2 ameliorates IRI damage

**Fig. 1:**
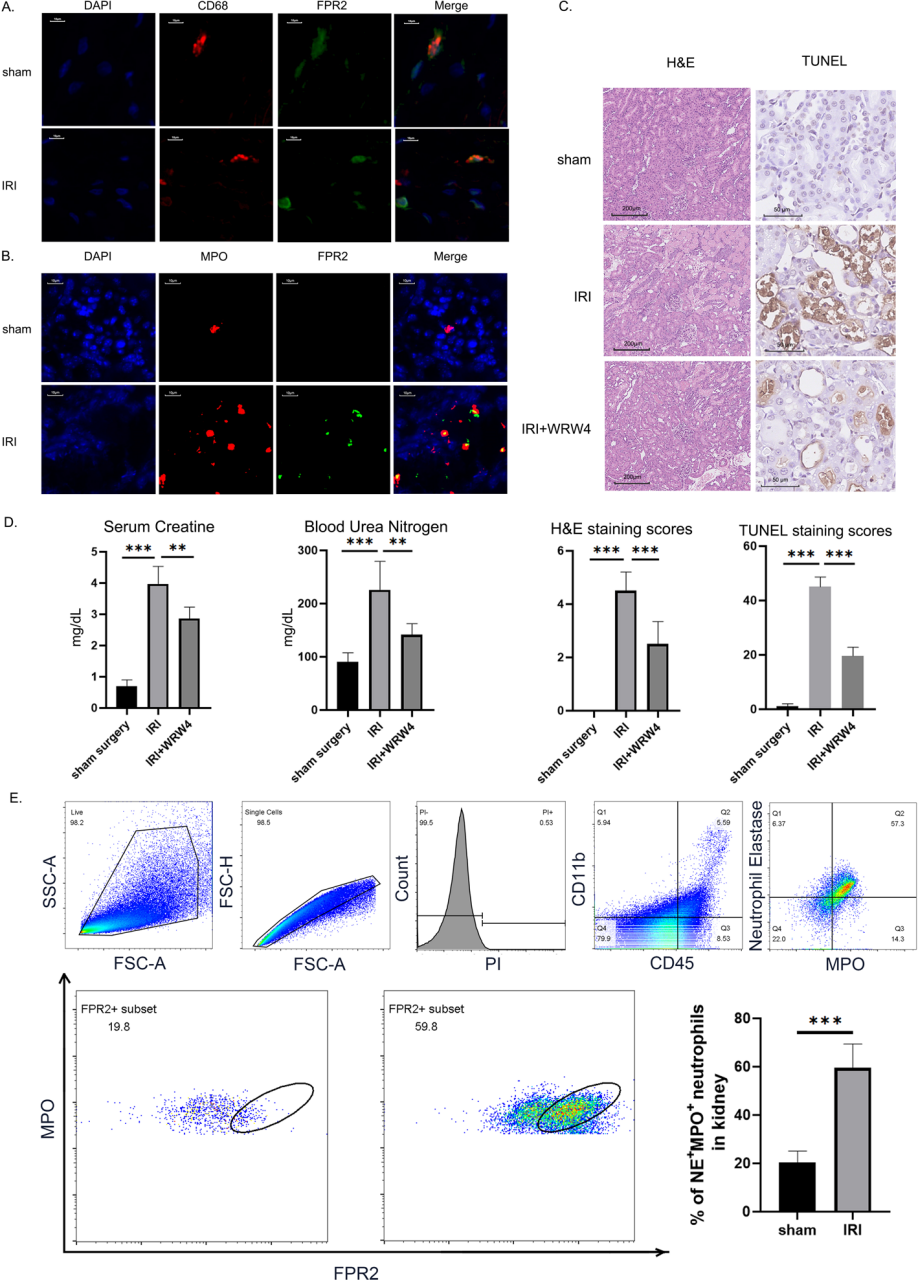
Inhibition of elevated FPR2 is protective against kidney injury. The left renal pedicle of the rat was clamped for 45 min to induce IRI. The right kidney was removed before releasing the clamps. WRW_4_ was administered intravenously 30 min before surgery. Serum or kidney tissues were collected 24 h after surgery. **A**, **B** Immunofluorescence staining of FPR2 colocalization with CD68^+^ macrophages and MPO^+^ neutrophils. Scale bar, 10 μm. Representative images from one experiment out of three are shown. **C** Kidney tissues were sectioned for histological examination and TUNEL staining. Scale bar, 100 μm. Representative images from one experiment out of three are shown. **D** Serum from sham surgery, IRI, and WRW_4_-treated rats was sampled at 24 h after surgery. Serum creatinine and blood urea nitrogen levels were measured. **E** The percentages of FPR2^+^ neutrophils in kidney tissues were determined by flow cytometry. The results are presented as the mean ± SEM from three independent experiments. **P < 0.01, ***P < 0.001

Rat kidneys were harvested 24 h after surgery. Alterations in FPR2 expression and localization in IRI kidneys were detected using immunofluorescence. We used CD68 to label macrophages and myeloperoxidase (MPO) to label neutrophils. FPR2 was expressed on macrophages in kidneys from rats in both the control and IRI groups, and the expression frequencies were similar between groups (Fig. 1A). However, on the surface of neutrophils, FPR2 showed marked expression only after IRI treatment (Fig. 1B). To quantitatively examine the trend of FPR2 expression in neutrophils, we analyzed neutrophils in kidney tissue using flow cytometry (Fig. 1E). The results of both sets of experiments were consistent in that the proportion of neutrophils expressing FPR2 increased significantly after IRI. This evidence suggests a correlation between FPR2 and IRI. However, the effect of elevated FPR2 expression on IRI outcome was still unclear.

To confirm the overall effects mediated by FPR2 in IRI, we preoperatively administered WRW_4_, a specific inhibitor of FPR2, via the tail vein to observe the alterations in IRI prognosis induced by FPR2 inhibition. We assessed morphological renal injury by observing H&E-stained sections and TUNEL-stained sections of rat kidneys and renal function changes by monitoring serum creatinine (Scr) and blood urea nitrogen (BUN) levels in rats.

Twenty-four hours after ischemia–reperfusion surgery, the rats in all groups were sacrificed to harvest peripheral blood and kidneys for further evaluation. We first evaluated renal injury from a pathological perspective. The harvested kidney tissues were stained with hematoxylin–eosin and then analyzed semi-quantitatively according to a pathology scoring system. The WRW_4_-pretreated kidneys had less proximal tubular injury, protein casts, and interstitial edema than the kidneys in the surgery group (Fig. 1C, quantified in Fig. 1D). TUNEL staining results showed a decrease in the number of apoptotic renal tubular cells in the WRW_4_ pretreatment group compared with the surgical group (Fig. 1C). These results suggest an ameliorative effect of FPR2 inhibition on ischemia–reperfusion-induced tissue injury. Next, we tested renal function parameters in peripheral blood. The rats showed a significant increase in both Scr and BUN levels, while WRW_4_ pretreatment protected against this injury (Fig. 1D). These results suggest a protective effect of FPR2 inhibition in ischemia–reperfusion-induced functional abnormalities.

Thus, specific inhibition of FPR2 during the acute phase of IRI is protective against both IRI-related tissue damage and functional abnormalities. This evidence suggests that FPR2 plays a pathological role by exacerbating injury during the acute phase of IRI. However, which upstream ligand signal FPR2 receives and through which cells it mediates this pathological effect still required further exploration.

### FPR2 inhibition reduced neutrophil infiltration

**Fig. 2:**
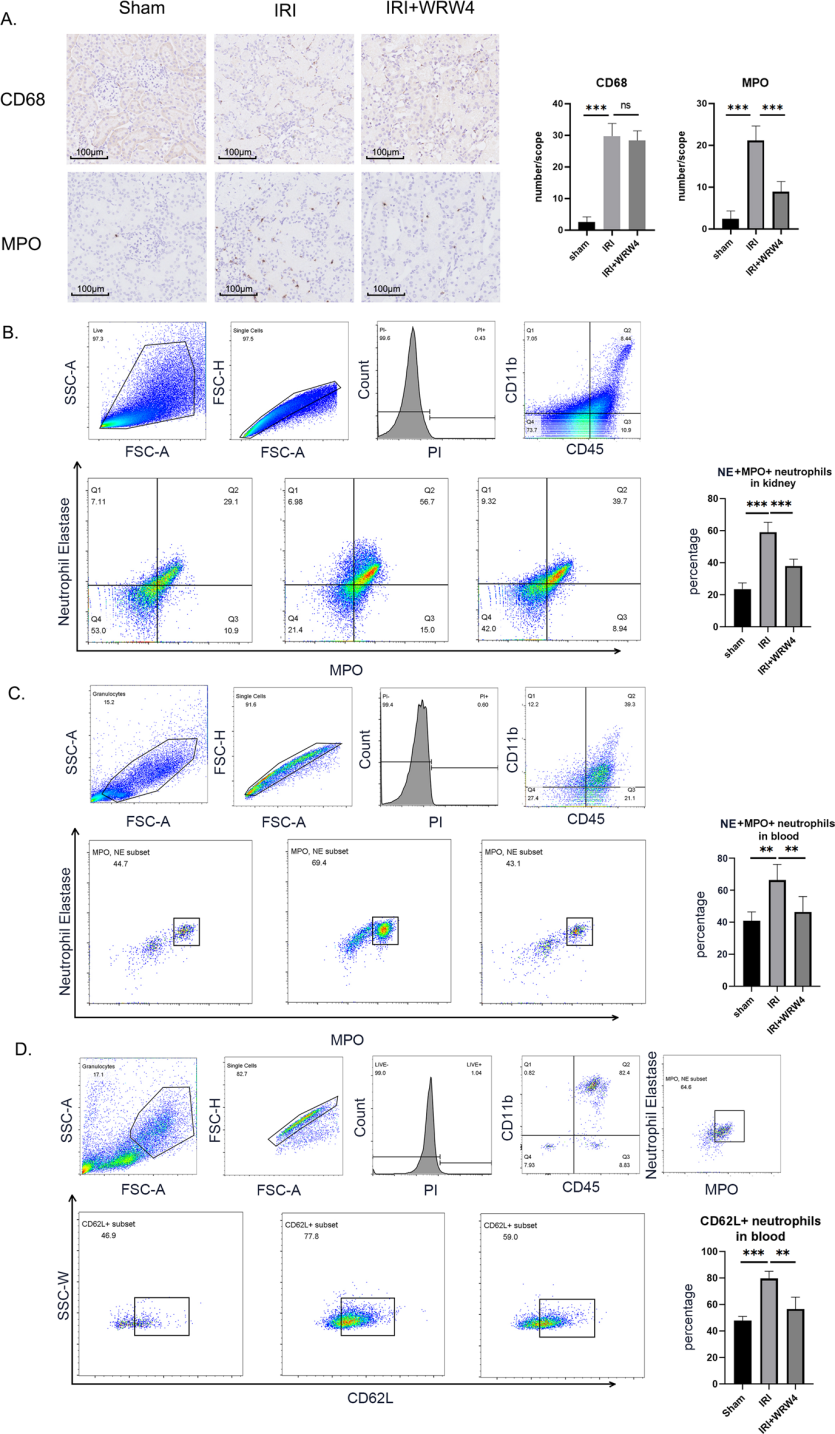
Inhibition of FPR2 reduces neutrophil infiltration in IRI kidneys. The left renal pedicle of the rat was clamped for 45 min to induce IRI. The right kidney was removed before releasing the clamps. WRW_4_ was administered intravenously 30 min before surgery. Kidney tissues and peripheral blood were collected 24 h after surgery. **A** Immunochemistry staining of CD68^+^ macrophages and MPO^+^ neutrophils. Scale bar, 100 μm. Representative images from one experiment out of three are shown. **B** The percentages of CD45^+^CD11b^+^MPO^+^NE^+^ neutrophils in kidney tissues were determined by flow cytometry. **C** The percentages of CD45^+^CD11b^+^MPO^+^NE^+^ neutrophils in peripheral blood were determined by flow cytometry. **D** Levels of CD62L on neutrophils in peripheral blood. The results are representative of three independent experiments. **P < 0.01, ***P < 0.001. ns, *P* > 0.05

On the basis of the results of immunofluorescence staining, FPR2 was found to be expressed on both macrophages and neutrophils, but which cell mediates the proinflammatory function of FPR2 in IRI was still unclear. The functions of FPR family molecules are usually characterized by the induction of immune cell migration. Therefore, we first examined kidneys from rats in the control, IRI, and WRW_4_-treated groups using immunohistochemistry to observe whether macrophage infiltration appeared to be altered after FPR2 inhibition. Although significant macrophage infiltration was confirmed in the kidneys of the IRI group, FPR2 inhibition did not reduce this infiltration. In contrast, immunohistochemical staining of neutrophils showed a significant decrease in neutrophil infiltration in ischemia–reperfused kidneys after FPR2 was specifically inhibited (Fig. 2A).

The results suggest that FPR2 exacerbates ischemia–reperfusion injury by mediating neutrophil infiltration during the acute phase of IRI.

We used flow cytometry to further examine the number of both kidney-infiltrating and peripheral blood neutrophils. Neutrophils were labeled using CD45, CD11b, neutrophil elastase (NE), and MPO. After IRI surgery, neutrophil counts were significantly elevated in both the kidney and peripheral blood of rats. However, WRW_4_ pretreatment blunted this neutrophil elevation (Fig. 2B, C). To test whether the migration ability of neutrophils to enter the lesion was elevated, we further examined the expression level of L-selectin (CD62L) on the surface of neutrophils in peripheral blood. CD62L expression on the surface of neutrophils was elevated after IRI surgery, and WRW_4_ pretreatment reduced CD62L levels (Fig. 2D).

### mtFPs are the main ligands of FPR2 in the acute phase of IRI

**Fig. 3:**
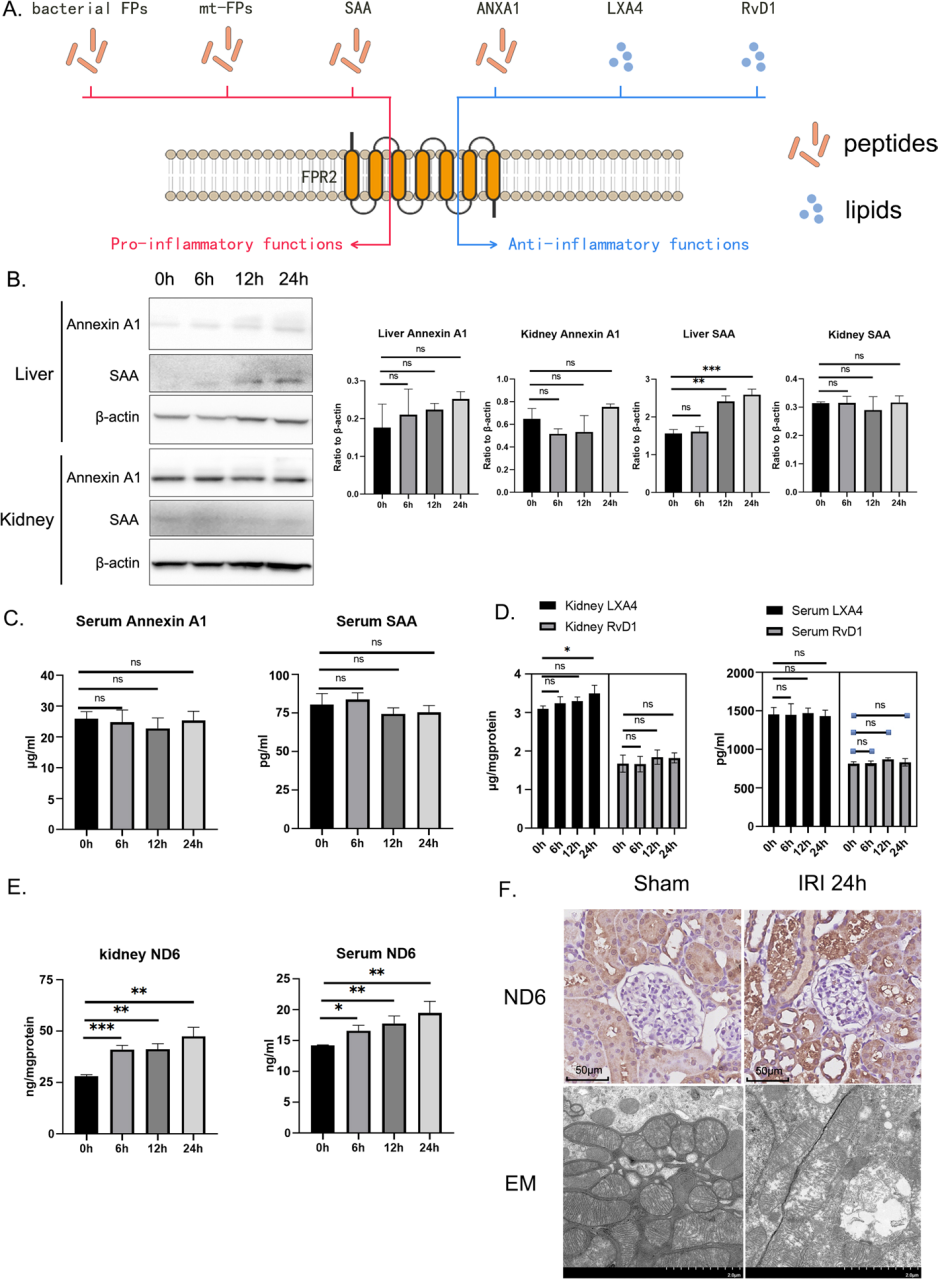
mtFPs are dominative FPR2 agonists in the acute phase of IRI. The left renal pedicle of the rat was clamped for 45 min to induce IRI. The right kidney was removed before releasing the clamps. WRW_4_ was administered intravenously 30 min before surgery. Liver tissues, kidney tissues, and peripheral blood were collected at 0, 6, 12, and 24 h after surgery. **A** Illustration of FPR2 agonists in a rat model. **B** At 0, 6, 12, and 24 h after surgery, the levels of ANXA_1_, SAA, and β-actin in IRI rat liver and kidney tissues were determined by western blot analysis. **C** At 0, 6, 12, and 24 h after surgery, the levels of ANXA_1_ and SAA in IRI rat serum were measured via enzyme-linked immunosorbent assay. **D** At 0, 6, 12, and 24 h after surgery, the levels of LXA_4_ and RvD1 in IRI rat kidney and serum were measured via enzyme-linked immunosorbent assay. **E** At 0, 6, 12, and 24 h after surgery, rat kidney and serum samples were collected. Kidneys were dissected and finely minced in Hank’s buffer containing 10% type IV collagenase, and the supernatant was collected as kidney interstitial tissues. Levels of ND_6_ in IRI rat kidney interstitial tissues and serum were measured via enzyme-linked immunosorbent assay. **F** At 0 and 24 h after surgery, rat kidneys were collected for immunochemistry staining of ND_6_ expression in kidneys and to obtain electron microscopy images of mitochondria in tubular cells. *P < 0.05, **P < 0.01, ***P < 0.001. ns, *P* > 0.05.

FPR2 is a functionally complex G-protein-coupled receptor and has multiple ligands, and these ligands produce different effects upon binding to FPR2. When the model animal is restricted to the rat, possible FPR2 ligands include bacteria-derived formyl peptides and mtFPs, as well as endogenous production of SAA, ANXA_1_, LXA_4_, and RvD1 (Fig. 3A). When the model is limited to aseptic inflammation, such as IRI, bacteria-derived formylated peptides should be excluded. We screened the remaining five FPR2 ligands in the kidney and peripheral blood of rats within 24 h postoperatively.

We first used western blotting and ELISAs to detect the protein ligands of FPR2. This class of ligands includes SAA and ANXA_1_. According to the available literature, the main source of SAA production is the liver. Therefore, we additionally harvested rat livers for tissue protein examination. Western blotting results showed that ANXA_1_ expression levels in the kidney did not change significantly at any timepoint within 24 h postoperatively (Fig. 3B). Although the expression levels of SAA in the liver appeared elevated at 12 h and 24 h postoperatively, the expression levels in the kidney did not change significantly at any postoperative timepoints in the sham-operated and IRI groups (Fig. 3B). We used ELISAs to detect the concentration of these two ligands in the serum and found no significant fluctuations in the serum concentrations of SAA and ANXA_1_ within 24 h after surgery (Fig. 3C). These two ligands are grouped because they both have the ability to attract neutrophils [24, 25], although ANXA_1_ is generally an anti-inflammatory FPR2 ligand [24]. However, since they do not appear to be significantly elevated in peripheral blood or kidneys during acute IRI, they are not the appropriate ligands to explain the elevated total number of neutrophils in peripheral blood and kidneys.

LXA_4_ and RvD1 are lipid FPR2 ligands produced in inflamed tissues. Therefore, we used ELISAs to detect changes in these two ligands in kidney and peripheral blood (Fig. 3D). Only LXA_4_ showed a progressive increasing tendency in the postoperative renal tissue and showed a significant difference compared with the control group at 24 h after surgery. However, we did not detect significant changes in LXA_4_ in peripheral blood. These results suggest that the anti-inflammatory FPR2 agonists LXA_4_ and RvD1 are not upregulated during the early phase of kidney IRI. Therefore, the inhibition of FPR2 in acute IRI is not hazardous for the natural healing process.

The characteristics of mtFPs are too vague to be directly detected; thus, we used the mitochondrial characteristic protein fragment ND_6_ as a representative molecule [26]. ND_6_ is one of the subunits of mitochondrial dehydrogenase. Mitochondrial dehydrogenase is expressed only inside the mitochondria and is released upon mitochondrial disruption, thus being recognized as a ligand for the formyl peptide receptor. Ischemia or ischemia–reperfusion injury can put tremendous stress on mitochondria and damage them. We used ELISAs to detect ND_6_ levels in the renal interstitium and peripheral blood. Compared with the control group, ND_6_ levels in the IRI group were significantly elevated at all timepoints within 24 h after surgery (Fig. 3E). Immunostaining of ND_6_ indicated an intense release of ND_6_ into interstitial tissue and peritubular capillaries, whereas ND_6_ was barely expressed if the kidney remained healthy. Under transmission electron microscopy, mitochondria in IRI kidneys were also damaged, characterized by vague cristae and shortened mitochondrial length [27] (Fig. 3F). These results indicate that the upregulated mtFPs are the main agonists of FPR2 during the early phase of kidney IRI. Damaged mitochondria in the kidney are the source of mtFPs. mtFPs are released from the mitochondria of cells into the intercellular space and then into the blood to activate peripheral neutrophils.

IRI is a complex pathological process that activates a variety of FPR2 ligands. SAA, LXA_4_, and mtFPs represented by ND_6_ all exhibit fluctuations in response to organismal injury. Considering that the inhibition of FPR2 during the acute phase of IRI produces significant therapeutic effects, we suggest that LXA_4_, an inflammatory ligand, is not a dominant ligand. Considering that SAA was not expressed in the kidney and that the peripheral blood changes were not significant, we believe that SAA is unlikely to explain the changes in renal injury status after FPR2 inhibition. The low expression of SAA in the kidney indicates its inability to direct the migration of FPR2-expressing immune cells to the lesion. In summary, the results revealed that mitochondrially derived peptides were the main agonists of FPR2 in the acute phase of IRI.

### mtFP triggers neutrophil proinflammatory responses through FPR2 in vitro

FPR2 is associated with the migration capacity of neutrophils and affects the total number of neutrophils infiltrating the kidney. In vitro experiments can exclude the confounding effect of quantity on overall cell function.

**Fig. 4:**
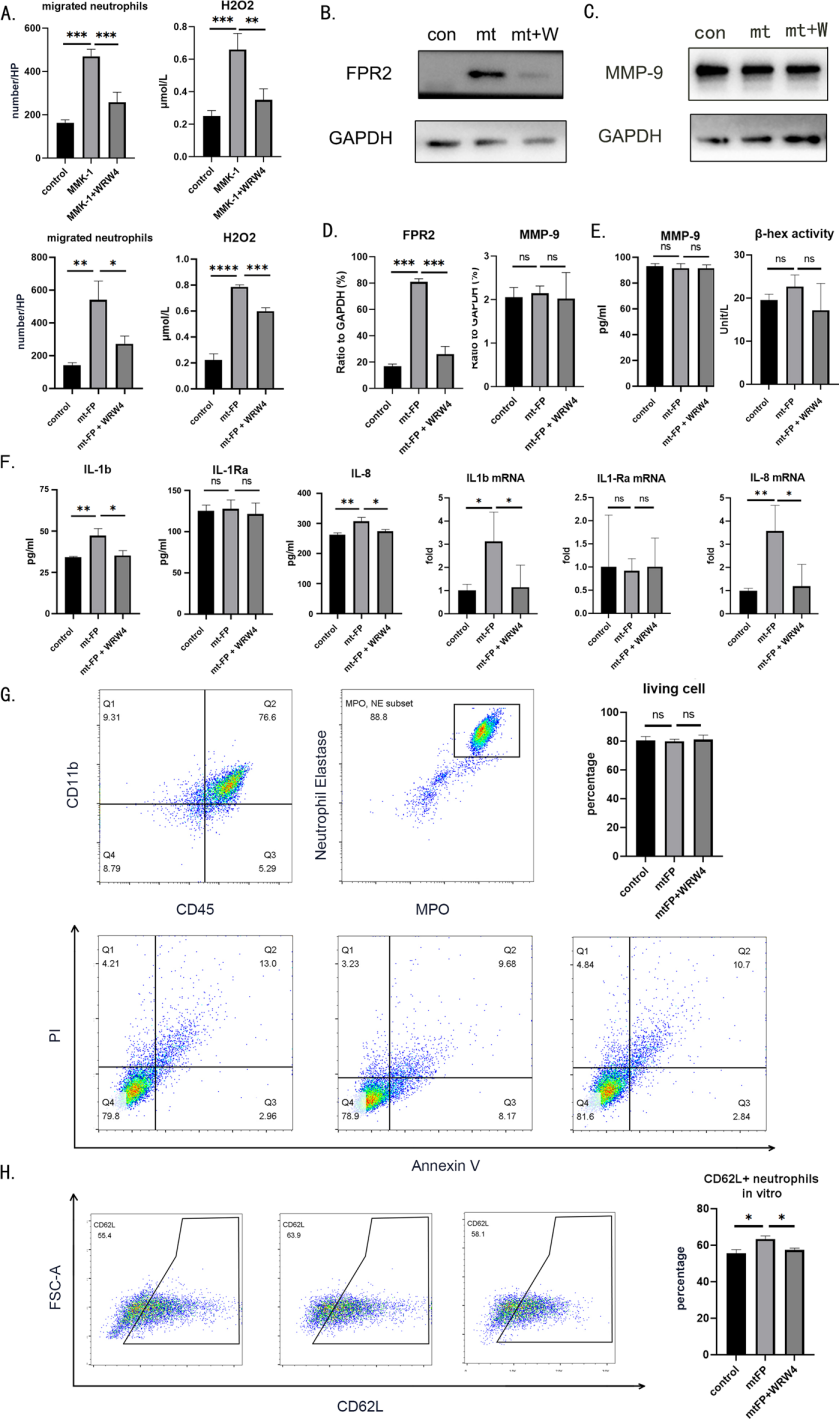
mtFP activates neutrophil proinflammatory responses through FPR2 in vitro. Primary neutrophils were purified from rat femur or tibia bone marrow using gradient centrifugation and cultured in pure RPMI 1640 medium. All in vitro experiments were conducted within 24 h after rat sacrifice. **A** Neutrophils were plated in the top chamber (pore size, 3 μm) of a transwell system. MMK-1 or mtFP was added to the bottom chambers, and the FPR2 inhibitor WRW_4_ was added to the top chambers. Two hours after top chamber insertion, migrated cells in the bottom chambers were counted. To measure H_2_O_2_ levels in cell culture medium, neutrophils were cocultured with MMK-1, MMK-1 + WRW_4_, mtFP, or mtFP + WRW_4_. After 2 h of coculture, the H_2_O_2_ concentration in the neutrophil culture medium supernatant was measured. **B** Neutrophils were cocultured with MMK-1 or MMK-1 + WRW_4_. After 2 h of coculture, neutrophils were extracted, and the levels of FPR2 and GAPDH were determined by western blot analysis (quantified in **D**). **C** Neutrophils were cocultured with mtFP or mtFP + WRW_4_. After 2 h of coculture, neutrophils were extracted, and the levels of MMP-9 and GAPDH were determined by western blot analysis (quantified in **D**). **E** Meanwhile, MMP-9 secretion and beta-hexosaminidase assays in supernatant were tested. **F** IL-1β, IL-1Ra, and IL-8 concentrations in supernatant were determined using enzyme-linked immunosorbent assays, with their intracellular mRNA expression levels determined by qPCR. **G** Neutrophils were cocultured with mtFP or mtFP + WRW_4_. After 2 h of coculture, neutrophils were extracted. Cell viability was measured using an Annexin V/PI assay and flow cytometry. **H** Neutrophils were cocultured with mtFP or mtFP + WRW_4_. After 2 h of coculture, neutrophils were extracted, and the expression of CD62L was measured via flow cytometry. The results are representative of three independent experiments. *P < 0.05, **P < 0.01, ***P < 0.001. ns, *P* > 0.05.

We first used MMK-1, a mitochondrially derived selective agonist of FPR2 [28], to confirm that FPR2 activation triggers neutrophil migration and ROS generation. The addition of MMK-1 to the lower chamber increased the number of neutrophils migrating downward from the upper chamber. The use of the FPR2-specific inhibitor WRW_4_ suppressed this migration (Fig. 4A). The H_2_O_2_ content in neutrophil culture supernatants can also be elevated by the addition of MMK-1. Correspondingly, inhibition of FPR2 using WRW_4_ reduced the H_2_O_2_ content in the supernatant (Fig. 4A). We then confirmed the correlation between mtFP and FPR2 in a primary neutrophil culture system. After extracting the cellular proteins, we examined the changes in FPR2 receptors using western blotting. The results showed that mtFP elevated FPR2 expression in neutrophils, while pretreatment with WRW_4_ attenuated this elevation (Fig. 4B).

We used transwell assays to detect the migratory capacity of neutrophils. The migration of neutrophils across the membrane was significantly increased by the induction of mtFP, while the addition of WRW_4_ attenuated the migration effect of mtFP (Fig. 4A). The addition of mtFP to the culture system induced neutrophils to release more hydrogen peroxide into the culture supernatant. Additional supplementation with WRW_4_ to inhibit FPR2 resulted in a definite inhibitory effect (Fig. 4A). Notably, neither intracellular production (Fig. 4C) nor extracellular secretion (Fig. 4E) of MMP-9 was affected by FPR2 activation or inhibition. We further examined the β-hexosaminidase assay in the culture supernatant and found no differences either (Fig. 4E). On the other hand, IL-1β and IL-8 levels in neutrophil culture supernatants were correlated with the activation and inhibition of FPR2, while IL-1Rα levels were not (Fig. 4F). This is consistent with the results of the in vivo experiments. To further confirm that the alteration in the number of neutrophils in the lesion was due to their migration capacity affected by FPR2, we examined changes in neutrophil viability in an in vitro experiment using an Annexin V-PI apoptosis assay. In these in vitro experiments, apoptosis or necrosis of neutrophils showed no correlation with activation or inhibition of FPR2 (Fig. 4G). At the same time, CD62L expression on the surface of neutrophils was upregulated by mtFP, and this change was abrogated by FPR2 inhibition using WRW_4_ (Fig. 4H). This further demonstrates that activation of FPR2 enhances the ability of cells to enter the lesion.

These results suggest that FPR2 on neutrophils can be activated by mtFP and inhibited by WRW_4_. Activation of FPR2 on neutrophils mediates neutrophil migration and ROS-dependent killing and alters the expression spectrum of inflammatory factors but does not alter the non-ROS-dependent killing capacity.

### FPR2 inhibition attenuates systemic and kidney inflammatory damage in IRI

**Fig. 5:**
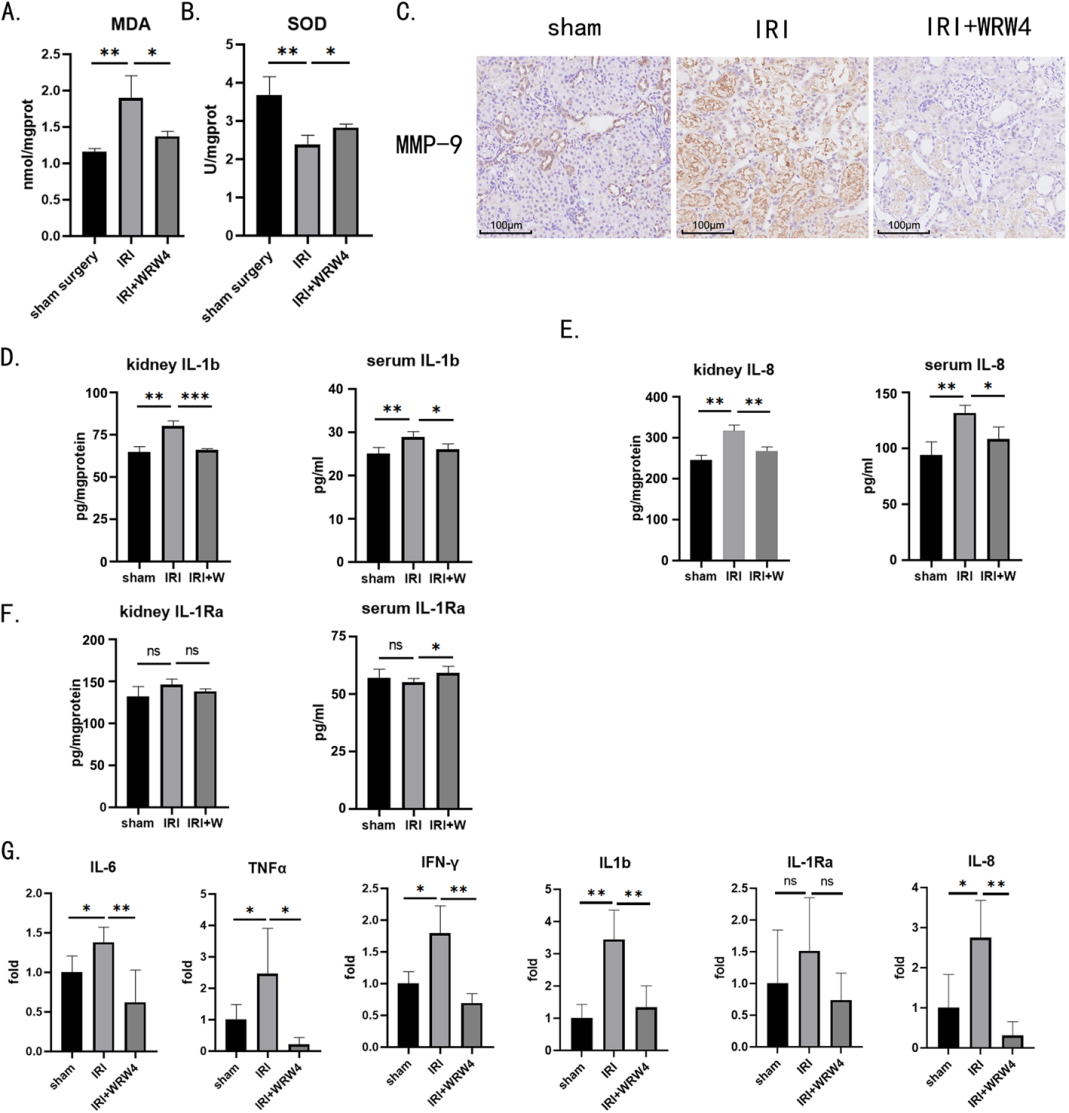
FPR2 inhibition alleviates systematic and local inflammation in IRI. The left renal pedicle of rats was clamped for 45 min to induce IRI. The right kidney was removed before releasing the clamps. WRW_4_ was administered intravenously 30 min before surgery. Kidney tissues and peripheral blood were collected 24 h after surgery. **A** MDA levels in rat kidneys were measured by thiobarbituric acid chromogenic reaction. **B** SOD levels in rat kidneys were measured by the WST-8 method. **C** Immunochemistry staining of MMP-9 expression in rat kidneys. Scale bar, 100 μm. Representative images from one experiment out of three are shown. **D**–**F** At 24 h after surgery, the levels of IL-1β, IL-1Ra, and IL-8 in rat kidneys and serum were measured using enzyme-linked immunosorbent assays. **G** At 24 h after surgery, the mRNA levels of IL-6, TNFα, IFN-γ IL-1β, IL-1Ra, and IL-8 in rat kidneys were measured via quantitative real-time PCR. Data are normalized to the expression levels in sham operation kidneys. *P < 0.05, **P < 0.01, ***P < 0.001. ns, *P* > 0.05

We have determined that activation and inhibition of FPR2 can affect neutrophil migration and reactive oxygen species production. Furthermore, we verified that a corresponding amelioration of renal injury could occur after in vivo administration of FPR2 inhibition. We found that, in IRI kidneys, MDA levels were significantly elevated compared with levels in the control group, while MDA levels in the WRW_4_-treated group were lower than those in the IRI group (Fig. 5A). Moreover, SOD activity was reduced in the kidneys of the IRI group, and the administration of WRW_4_ pretreatment rescued some SOD activity (Fig. 5B). We further examined non-ROS-dependent injury. MMP-9 was chosen as a molecular indicator of relative IRI damage [29]. After immunohistochemical staining of kidney tissue sections, we found that MMP-9 was deeply stained in the kidneys of rats in the IRI group, and the application of WRW_4_ reduced the level of MMP-9 (Fig. 5C). Finally, we examined changes in the concentrations of several inflammatory cytokines in the kidney and peripheral blood. IL-1β and IL-8 are proinflammatory cytokines that activate immune cells and chemoattract immune cells, respectively, while IL-1Rα is a natural inhibitor of IL-1β and has an inhibitory effect on immune cell activation. The results suggested that the expression levels of both IL-1β and IL-8 were elevated by IRI and decreased by WRW_4_ treatment in both kidney and peripheral blood (Fig. 5D and E). In contrast, IL-1Rα was not affected (Fig. 5F). Global and local inflammation levels in the kidney also appeared to be reduced by WRW_4_ intervention. Meanwhile, the mRNA expression levels of typical cytokines in IRI, including IL-6, TNFα, and IFN-γ, together with IL-1β, IL-1Rα, and IL-8 in the kidney were elevated after IRI treatment, whereas WRW_4_ pretreatment reduced the expression levels of these mRNAs (Fig. 5G).

These results demonstrate that both systematic and kidney inflammation are alleviated after neutrophil FPR2 inhibition, indicating curative effects of WRW_4_ intervention on kidney IRI.

### The ERK1/2 pathway mediates mtFP–FPR2 axis-activated neutrophil proinflammatory functions in vitro

**Fig. 6:**
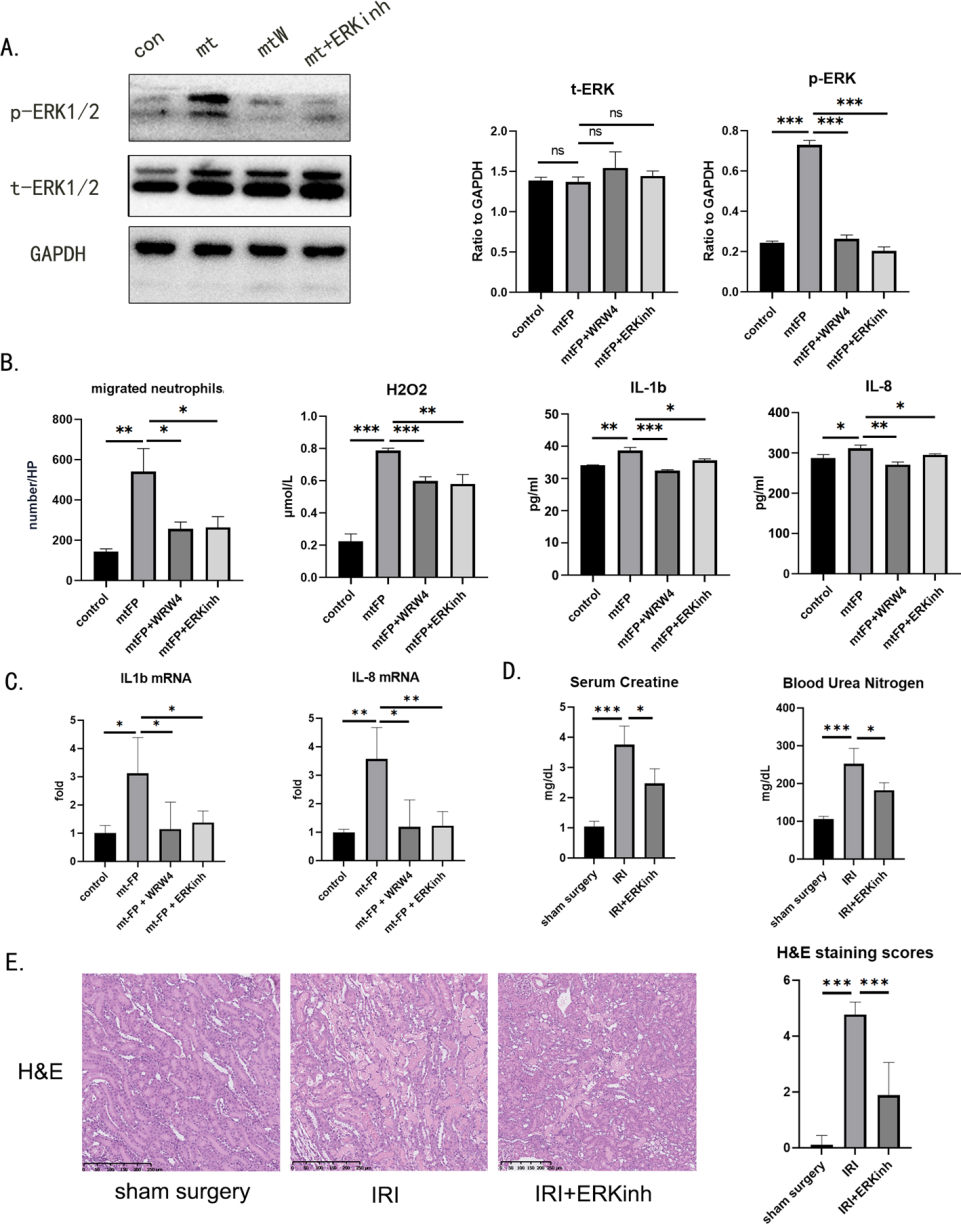
The ERK1/2 pathway mediates mtFP–FPR2 axis-activated neutrophil proinflammatory functions. Primary neutrophils were purified from rat femur or tibia bone marrow using gradient centrifugation and cultured in pure RPMI 1640 medium. All in vitro experiments were conducted within 24 h after rat sacrifice. **A** Neutrophils were cocultured with mtFP, mtFP + WRW_4_, or mtFP + SCH772984. After 2 h of coculture, the expression levels of p-ERK1/2, t-ERK1/2, and GAPDH in neutrophils were determined by western blot analysis. **B** Neutrophils were plated in the top chamber (pore size, 3 μm) of a transwell system. Synthesized mitochondrial peptide mtFP were added to the bottom chambers, and the FPR2 inhibitor WRW_4_ or the ERK1/2 pathway inhibitor SCH772984 was added to the top chambers. Two hours after top chamber insertion, migrated cells in the bottom chambers were counted. To measure H_2_O_2_ levels in cell culture medium, neutrophils were cocultured with mtFP, mtFP + WRW_4_, or mtFP + SCH772984. After 2 h of coculture, the H_2_O_2_ concentration in the neutrophil culture medium supernatant was measured by the Fenton reaction method. **C** Meanwhile, the levels of IL-1β and IL-8 in the supernatant were determined by enzyme-linked immunosorbent assays. **D**, **E** Serum from sham surgery, IRI, and SCH772984-treated rats was sampled at 24 h after surgery. Serum creatinine and blood urea nitrogen levels were measured. Kidney tissues were sectioned for histological examination. Scale bar, 100 μm. *P < 0.05, **P < 0.01, ***P < 0.001. ns, *P* > 0.05

To investigate the mechanism by which FPR2 affects neutrophils, we examined intracellular ERK1/2 and phosphorylated ERK1/2 levels in neutrophils using western blotting. The ERK1/2 pathway is an intracellular signaling pathway that is often activated by the FPR family. We successfully verified the activation of ERK1/2 phosphorylation in mtFP-induced neutrophils and further added the ERK inhibitor SCH772984 to verify whether downstream function was affected (Fig. 6A). When activation of the ERK1/2 pathway was inhibited with SCH772984, neutrophil immune functions that could have been activated by mtFP, including cell migration, ROS-dependent killing, and changes in cytokine expression, were all suppressed (Fig. 6B, C). We further injected ERKINH into rats through tail vein injection 30 min before surgery and confirmed the protective effect of ERK inhibition on renal IRI in vivo by serum creatinine, urea nitrogen, and H&E section stainings (Fig. 6D-E).

These results suggest that the domination of the mtFP–FPR2 axis over downstream functions of neutrophils is dependent on the activation (indicated by phosphorylation) of the intracellular ERK1/2 pathway.

## Discussion

Our findings confirm that mitochondria-derived formyl peptides activate neutrophil FPR2 and exacerbate kidney IRI damage. Preapplication of WRW_4_ can antagonize FPR2 and block the activation of mtFP on neutrophils in the early phase of IRI, thus alleviating the damage and inflammation caused by kidney IRI. The ERK1/2 pathway is a key pathway by which FPR2 activates immune function downstream of neutrophils.

FPR2 is expressed on the surface of a variety of cells and mediates a variety of cellular functions [9]. FPR2 was first found to be expressed on the surface of myeloid immune cells, such as neutrophils, macrophages, and dendritic cells [30]. FPR2 expression can also appear on the surface of T cells and B cells [30]. Recent studies have found that tissue cells, such as alveolar epithelial cells and vascular endothelial cells, can also express FPR2 and mediate disease progression or healing through FPR2 [19, 31]. In the kidney, Only fibroblasts have been reported to express FPR2 [32]. We therefore attribute the curative effects of WRW_4_, an FPR2-specific inhibitor, to its influence on systemic or local immune cells in the kidney. Among the various types of immune cells, we chose neutrophils and macrophages as the preferred screening targets for the following reasons. First, FPR2 is reliably expressed on neutrophils and macrophages. In contrast, in the case of CD4^+^ T cells, for example, the expression of FPR family receptors on the surface of these T cells is not stable and varies with activation conditions and disease conditions [33]. Second, neutrophils and macrophages are classical immune cells involved in IRI injury and have a direct and precise effect [34]. Finally, and most importantly, in our preliminary experiments, the immunofluorescence results showed that most of the FPR2-positive cells in the renal interstitium were costained by fluorescent markers of neutrophils and macrophages. Our experimental results showed that the number of neutrophil infiltrates in the kidney decreased remarkably after WRW_4_ treatment, whereas the change in the total number of macrophages did not reach a significant difference. Considering that many studies on macrophage FPR2 have stressed the subphenotype changes of macrophages [35, 36], we also examined the phenotyping of kidney macrophages after WRW_4_ treatment (Additional file 1: Fig. S1). However, no significant change was observed in the absolute number of macrophages or in the proportion of either M1 or M2 type macrophages among the total number of macrophages. Therefore, we believe that the therapeutic effect produced by FPR2 inhibition is most reliably explained by changes in neutrophils. It is true that simple subphenotyping cannot completely define the cell functional changes of macrophages and cannot completely exclude the possibility of macrophage involvement in IRI [37]. FPR2 is a multifaceted receptor that mediates important cellular functions, and more detailed studies are still needed to address the effects of this receptor on other cellular functions in IRI.

In the FPR family, the distinctive anti-inflammatory properties of the FPR2 receptor are often used as central features in research [24, 38, 39]. The fundamental ability of FPR2 itself to recognize formylated peptides is often neglected. It has been revealed that the concentration threshold for the activation of FPR2 by specific formyl peptides is significantly lower than that of FPR1 [40]. In studies investigating FPR2 ligands, it has been noted that some mitochondrially derived formyl peptides bind more strongly to FPR2 than to FPR1 [40, 41]. In our initial explorations, we were not sure what role FPR2 plays in IRI. There are multiple agonists of FPR2, and all have relatively clear proinflammatory or anti-inflammatory effects and thus are not suitable for the exploration of natural disease mechanisms. In contrast, the FPR2-specific inhibitor WRW_4_ can be used to determine how the prognosis of IRI is altered by diminished FPR2 function. In FPR2 studies that focus on organ IRI, investigators often use FPR2 as a therapeutic target and administer ligands with anti-inflammatory effects to explore therapeutic effects and underlying mechanisms [35, 38]. We also expected that FPR2 inhibition would cause an increase rather than a decrease in IRI injury, but after WRW_4_ application, we observed a therapeutic effect that, although not very dramatic, could not be ignored. Such data forced us to revisit the characteristics of FPR2 as a receptor and reminded us that disease progression and the function of the receptor were not static. Our data suggest that FPR2 mediates the exacerbation of IRI in the 24 h following IRI. It has been reported in the literature that FPR2 mediates the beneficial functions of tissue repair and inflammation dissipation [35, 42]. These studies focused on different timepoints in the disease, with few reports on the acute phase. After limiting the duration of disease investigation to 24 h after surgery, we validated possible FPR2 ligands on a case-by-case basis and found that mtFPs are the primary FPR2 ligands 24 h after IRI and mediate the exacerbation of inflammation through FPR2. In addition, it is noteworthy that our experimental results showed that LXA_4_ exhibited a gradual increase 24 h after IRI. This may suggest that the repair mechanism related to FPR2 is being initiated in the kidney.

Under inflammatory conditions, formylated peptides are important guiding molecules for immune cell chemotaxis and migration. The classical view is that formylated peptides guide immune cells after penetrating the endothelium, leaving the endothelium and finding the lesion of injury [43]. Formyl peptides are considered the last route of concentration gradient-guided chemotaxis. However, in recent years, it has been documented that there is a significant rise in formylated peptides in peripheral blood under conditions of injury [44]. Our results show that mtFPs are present in peripheral blood under IRI conditions. Additionally, mtFPs in peripheral blood can enhance CD62L (L-selectin) expression in neutrophils, which might enhance the ability of neutrophils to penetrate the endothelium and enter the lesion.

The diversity and complexity of FPR2 ligands are well recognized. Five ligands (mtFPs, ANXA_1_, SAA, LXA_4_ and RvD1) were selected for our study. Some of the FPR2 ligands were reasonably excluded on the basis of the available literature. For example, bacteria-derived formyl peptides are not involved in sterile IRI injury. These ligands are often the research focus in FPR2-related studies. On the other hand, in our study, these possible FPR2 agonists were screened, providing a reference for related studies. The therapeutic role of ANXA_1_, LXA_4_, and RvD1 in inflammatory diseases, such as IRI, is well recognized, but in recent years, there have been few studies on their natural expression in diseases. In chronic diseases, such as diabetic nephropathy, the level of ANXA_1_ expression in the kidney tissue of patients is significantly increased [38]. In our study, ANXA_1_ was not significantly upregulated in the kidney. This finding might be related to the earlier time point of injury that we selected. On the other hand, LXA_4_ showed a potential upward trend, which may suggest a gradual initiation of the repair process in the kidney. Further study of the natural trends of these ligands will help in understanding the FPR2-associated damage/repair patterns in organisms and in determining the appropriate timing of FPR2 inhibition.

Molecular isoforms of genes, proteins, and other living substances that perform the same function often vary between species and consequently affect the translation of experimental findings. Our study has similar limitations. Although the ligands included in the experiments have a fairly reliable basis for translation, the possibility that FPR2 could have ligands in rats that are not present in humans cannot be excluded. The inhibitory effect of WRW_4_ may also not be limited to FPR2 as an FPR family receptor. In mice alone, the FPR family has nine different receptor forms, of which only some FPR molecules correspond to human FPR [38]. Related molecular biology studies remain to be further explored. However, mtFP molecules retain fairly high homology among animals. From this perspective, our findings have good translation potential.

Neutrophils are important effector cells of the intrinsic immune system. Neutrophils remove invading pathogens and damaged cells through migration, phagocytosis, and ROS-dependent and non-ROS-dependent killing functions. Neutrophils also have an ability to secrete cytokines. In IRI, the manner in which FPR2-activated neutrophils damage tissue is still poorly understood. The bacteria-derived peptide PSMα can bind to FPR2 on neutrophils, but its downstream activation only activates NADPH oxidase activity, producing ROS-related killing effects [45]. In another study, activation of FPR2 on neutrophils altered the expression of certain chemokines, such as CCR2 and CXCR4, and triggered changes in the expression of the inflammatory factor IL-6 [46]. In our study, neutrophils activated FPR2 downstream functions after coculture with a reported mitochondrially derived selective agonist of FPR2 [28], and FPR2 expression was elevated. This is consistent with the previous findings of Andrea et al. [46]. The addition of mtFP activated the migratory capacity, ROS-dependent killing capacity and inflammatory cytokine expression of neutrophils, altering neutrophil function in a proinflammatory direction. These results suggest that activation of neutrophils by mtFPs exacerbates injury by increasing the number of neutrophil infiltrates in the kidney and by enhancing their oxidative burst levels. The degranulation response of neutrophils is an important pathway for generating tissue damage. fMLP activates neutrophils upon binding to FPR1 and can enhance their degranulation response[47]. However, in our study, activation of FPR2 by mtFP did not produce an increase in MMP-9 synthesis or secretion. This may be related to the fact that there are differences in the receptor-mediated functions of FPR family members. Our results reveal that the increase in ROS-related and non-ROS-related killing in neutrophils stimulated by mtFP is not synchronized. Xu et al. [48] suggest that a rise in ROS levels within neutrophils suppresses the level of degranulation, which is consistent with our findings. Our combined use of specific inhibitors of FPR1 and FPR2 in rat kidney IRI revealed no significantly better renal function and tissue damage compared with FPR2 inhibition alone, although inflammation was reduced (Additional file 2: Fig. S2).

IRI is an inflammatory response that mixes multiple pathological processes. Oxidative stress is one of the essential features of IRI, while matrix-degrading enzymes, such as MMP-9, that are secreted by neutrophils and other cells promote the spread of inflammation. A recent study showed that gadolinium chloride inhibits superoxide anion production in neutrophils in vitro and promotes apoptosis [49]. In vivo, the ameliorative effect of gadolinium chloride on colitis was manifested by a decrease in local tissue MDA levels and an improvement in total SOD activity [49]. In a model of pulmonary fibrosis, neutrophils were found to secrete characteristic NE and MMP-9 and other factors that exacerbate lung inflammation. Treatment with gingival-derived mesenchymal stem cells may improve lung prognosis by reducing lung oxidative stress and the expression levels of NE and MMP-9 [50]. Our data are consistent with these studies. After confirming that WRW_4_ inhibits neutrophil migration and oxidative damage, we observed a reduction in the associated inflammatory damage in an in vivo model. Notably, in vitro experiments indicated that neutrophils did not exhibit reduced synthesis or secretion of MMP-9 in response to FPR2 inhibition, but in vivo experiments showed a significant reduction in neutrophil-dominant MMP-9 secretion. We consider this to be the result of a decrease in the total number of infiltrating neutrophils. In IRI models, early massive neutrophil infiltration is often considered the hallmark event of injury [51]. At this point, the repair function of neutrophil clearance of dead cells is secondary to the side effects of tissue injury [51]. It is beneficial to reduce neutrophil infiltration during this period. The local and global decrease in inflammation was also corroborated by changes in the expression profile of inflammatory factors in our study.

The ERK1/2 pathway is involved in downstream FPR2 signaling. FPR2 expressed in human lung fibroblasts can be activated by ANXA_1_ and attenuate TGFβ-induced fibrosis and inflammatory tendencies through the ERK1/2 pathway [52]. Upon binding to the proinflammatory ligand SAA, FPR2 on the macrophage surface also initiates downstream molecular expression via the ERK1/2 pathway [53]. Our results are consistent with these previous studies, showing that mtFP binding to FPR2 also activates downstream cellular functions via the ERK1/2 pathway, which can be inhibited by inhibiting phosphorylation of ERK1/2. According to the existing findings, the ERK1/2 pathway is an important pathway downstream of FPR2. FPR2 can also activate other downstream pathways, such as the PI3K/Akt pathway and mTOR pathway [35, 54]. Relying on only one pathway, ERK1/2, may not explain the complex functions mediated by FPR2. In addition, we noted that the specific inhibition of FPR2 did not achieve complete inhibition of ERK1/2 pathway phosphorylation. This may be related to the simultaneous activation of FPR1 by mtFPs, which also induces activation of the downstream ERK1/2 signaling pathway [55]. The combination of cyclosporine H, specific inhibitors of FPR1, and WRW_4_ can better reduce the phosphorylation level of ERK1/2.

## Conclusions

This study demonstrated that the mtFP–FPR2 axis activates neutrophil immune function through the ERK1/2 signaling pathway and exacerbates renal injury during the acute phase of renal IRI. These findings suggest that presuppression of FPR2 in the acute phase of IRI may improve renal prognosis and further confirm that FPR2 is important in promoting inflammation.

## Supplementary Information

**Additional file 1: Fig. S1.** Macrophage infiltration and fractionation were unaffected by FPR2 inhibition. Infiltrated macrophages were labeled using CD45 and CD68, and then subpopulated with CD86 and CD163. *P < 0.05, **P < 0.01, ***P < 0.001. ns, *P* > 0.05.

**Additional file 2: Fig. S2.** Combination of FPR1 and FPR2 inhibitors improves inflammation, but efficacy improvement is not significant. (**A**, **B**) Kidney tissues were sectioned for histological examination. Scale bar, 100 μm. Representative images from one experiment out of three are shown. (**C**) Serum from sham surgery, IRI, WRW4-treated, and CsH/WRW4-treated rats was sampled at 24 h after surgery. Serum creatinine and blood urea nitrogen levels were measured. (**D**) MDA levels in rat kidneys were measured by thiobarbituric acid chromogenic reaction, and SOD levels in rat kidneys were measured by the WST-8 method. (**E**) At 24 h after surgery, the mRNA levels of IL-6, TNFα, and IFN-γ in rat kidneys were measured via quantitative real-time PCR. Data are normalized to the expression levels in sham operation kidneys. *P < 0.05, **P < 0.01, ***P < 0.001. ns, *P* > 0.05.

## Data Availability

The datasets used and/or analyzed during the current study are available from the corresponding author on reasonable request.

## Abbreviations

IRI: Ischemia–reperfusion injury
FPR2: Formyl peptide receptor 2
mtFPs: Mitochondrial formyl peptides
ERK: Extracellular regulated protein kinases
DGF: Delayed graft function
PAMPs: Pathogen-associated molecular patterns
DAMPs: Damage-associated molecular patterns
ROS: Reactive oxygen species
ANXA1: Annexin A1
RvD1: Resovin D1
LXA4: Lipoxin A4
SAA: Serum amyloid A
ND6: NADH dehydrogenase subunit 6
ELISA: Enzyme-linked immunosorbent assay
MDA: Malondialdehyde
SOD: Superoxide dismutase
MPO: Myeloperoxidase
TUNEL: Terminal deoxynucleotidyl transferase-mediated dUTP nick end labeling
Scr: Serum creatinine
BUN: Blood urea nitrogen
MMP-9: Matrix metalloproteinase-9
