# Supplementary figures and images for: Formyl peptide receptor 2 activation by mitochondrial formyl peptides stimulates the neutrophil proinflammatory response via the ERK pathway and exacerbates ischemia–reperfusion injury
Source: Cell Mol Biol Lett. 2023 Jan 19;28:4. doi: 10.1186/s11658-023-00416-1 (PMC9854225; doi:10.1186/s11658-023-00416-1)

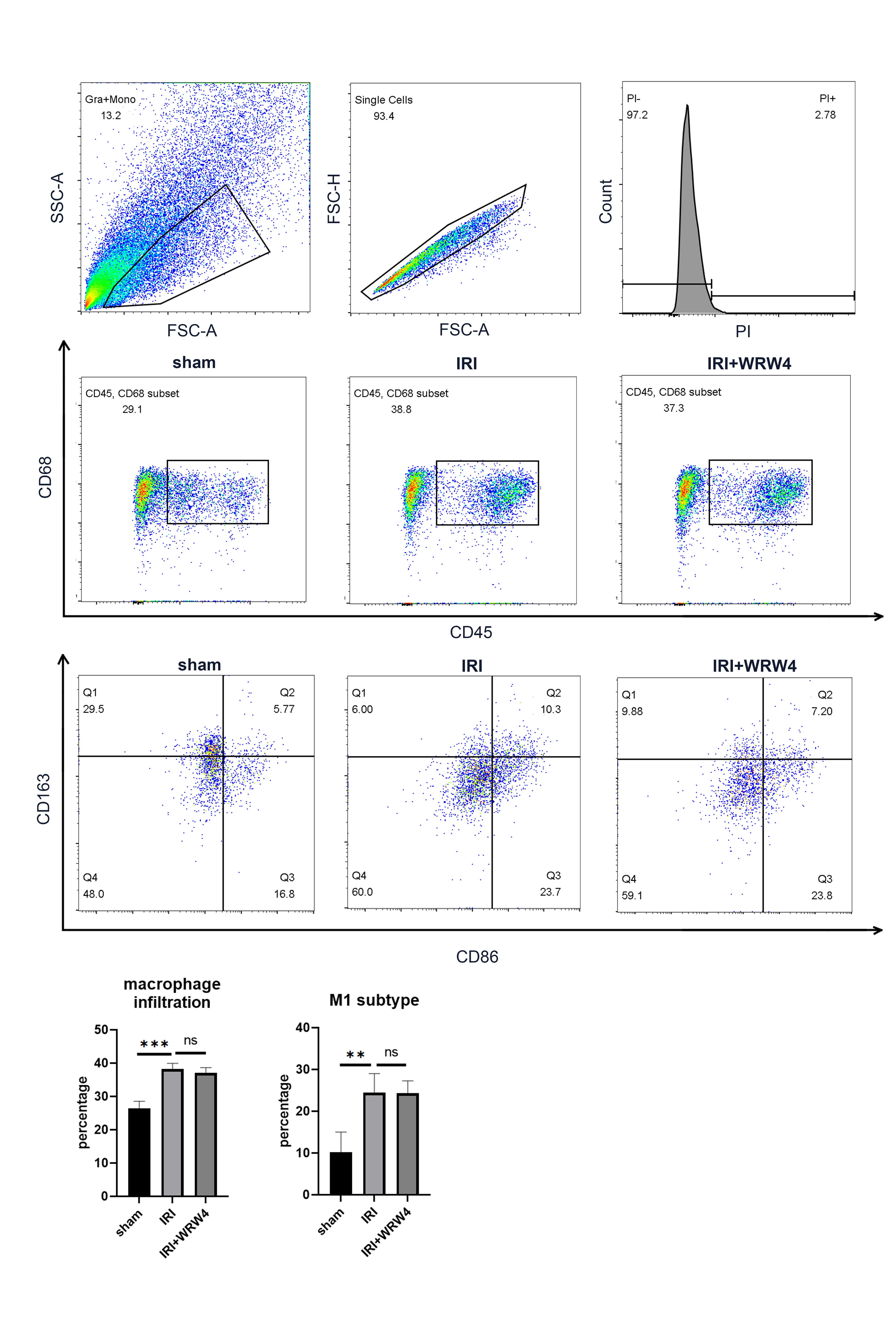

Supplement: Supplementary file 1 — Additional file 1: Fig. S1. Macrophage infiltration and fractionation were unaffected by FPR2 inhibition. Infiltrated macrophages were labeled using CD45 and CD68, and then subpopulated with CD86 and CD163. *P < 0.05, **P < 0.01, ***P < 0.001. ns, P > 0.05. [file 11658_2023_416_MOESM1_ESM.jpg]

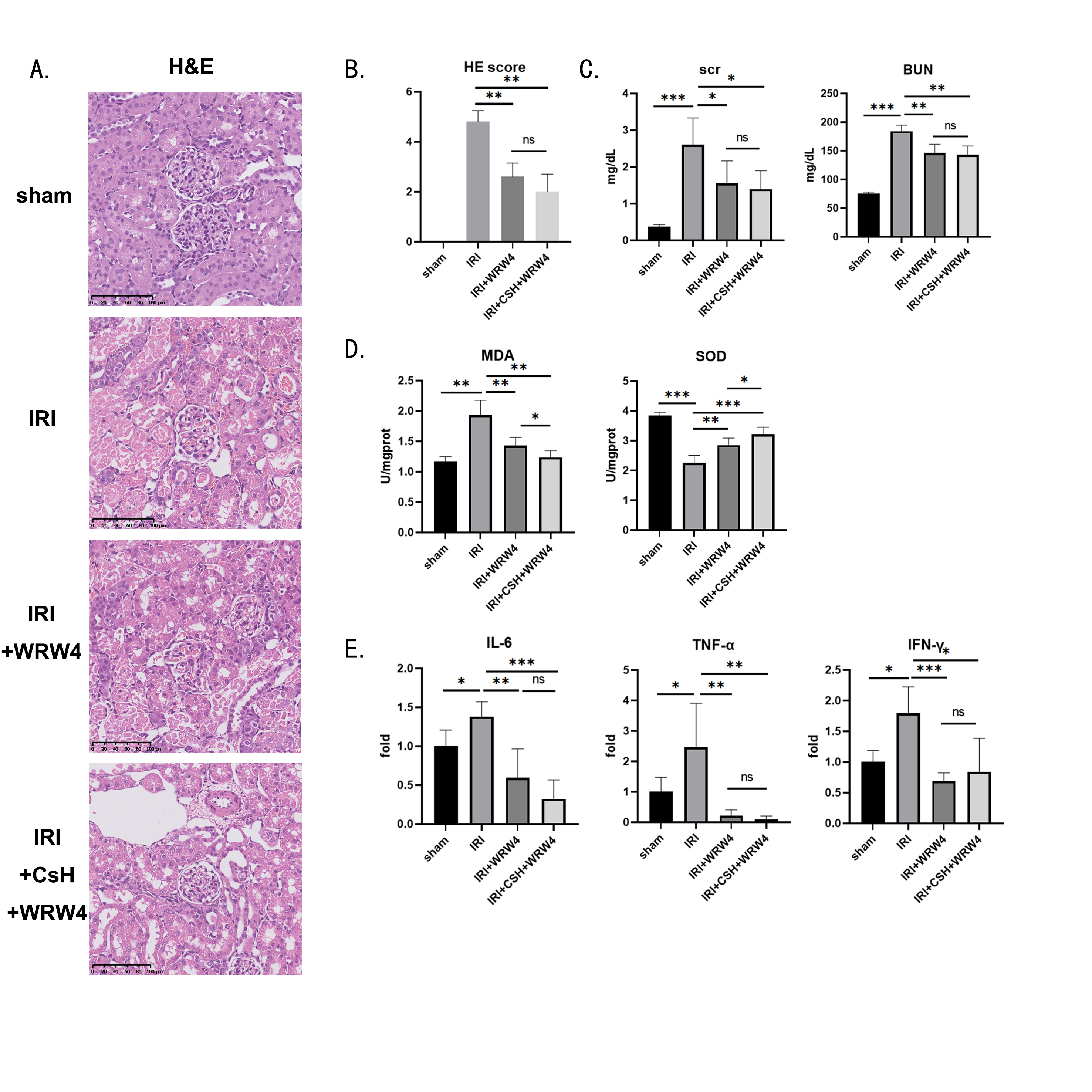

Supplement: Supplementary file 2 — Additional file 2: Fig. S2. Combination of FPR1 and FPR2 inhibitors improves inflammation, but efficacy improvement is not significant. (A, B) Kidney tissues were sectioned for histological examination. Scale bar, 100 μm. Representative images from one experiment out of three are shown. (C) Serum from sham surgery, IRI, WRW4-treated, and CsH/WRW4-treated rats was sampled at 24 h after surgery. Serum creatinine and blood urea nitrogen levels were measured. (D) MDA levels in rat kidneys were measured by thiobarbituric acid chromogenic reaction, and SOD levels in rat kidneys were measured by the WST-8 method. (E) At 24 h after surgery, the mRNA levels of IL-6, TNFα, and IFN-γ in rat kidneys were measured via quantitative real-time PCR. Data are normalized to the expression levels in sham operation kidneys. *P < 0.05, **P < 0.01, ***P < 0.001. ns, P > 0.05. [file 11658_2023_416_MOESM2_ESM.jpg]
